# Closeness to friends explains age differences in positive emotional experience during the lockdown period of COVID-19 pandemic

**DOI:** 10.1007/s40520-021-01927-7

**Published:** 2021-07-11

**Authors:** Elena Cavallini, Alessia Rosi, Floris Tijmen van Vugt, Irene Ceccato, Filippo Rapisarda, Martine Vallarino, Luca Ronchi, Tomaso Vecchi, Serena Lecce

**Affiliations:** 1 Department of Brain and Behavioral Sciences, University of Pavia, Piazza Botta 6, 27100 Pavia, Italy; 2 Department of Psychology, University of Montreal, Montreal, QC Canada; 3 Deparment of Neuroscience, Imaging and Clinical Sciences, University G. d’Annunzio of Chieti-Pescara, Chieti, Italy; 4 Sociosfera ONLUS SCS, Seregno, Italy; 5 Cognitive Psychology Unit, IRCCS Mondino Foundation, Pavia, Italy

**Keywords:** Positive emotion, Negative emotion, COVID-19, Closeness, Activity

## Abstract

**Background:**

Studies on age differences in emotional states during the COVID-19 pandemic showed that older adults experienced greater emotional wellbeing compared to younger adults. We hypothesized these age differences to be related to the perception of closeness to family/friends or the engagement in daily activities during the pandemic.

**Aim:**

To investigate age differences in positive and negative emotional experiences and whether the perception of closeness to family/friends and the engagement in daily activities during pandemic explained such age-related differences.

**Methods:**

Through a cross-sectional study, 1,457 adults aged 18–87 years old completed an online survey assessing positive and negative emotional experiences, the perception of more closeness to family/friends, and daily activities that participants started/re-started during the pandemic.

**Results:**

Increasing age was associated with more positive and less negative emotional experiences. Age differences in positive emotional experience were explained by the perception of more closeness to friends and not by the engagement in daily activities. For negative emotional experience age, differences remained significant even after accounting for the perception of closeness to family/friends and engagements in daily activities.

**Discussion:**

Older adults’ greater overall level of positive emotional experience was explained by their greater perception of more closeness to friends. We speculate that social closeness provides a coping mechanism to increase emotional wellbeing employed especially in older adults.

**Conclusion:**

Our findings reinforce the link between perceived social closeness and emotional wellbeing especially in older adults. To cope with stressful situation, it is important to encourage older adults to increase the closeness to their social network.

## Introduction

The spread of COVID-19 pandemic provided the opportunity for researchers to answer several questions that experimentally would not be possible to investigate, such as the emotional impact in aging during a prolonged period of isolation and the resulting stress and concern. Research conducted during the early phase of the COVID-19 outbreak reported that older adults exhibited greater emotional wellbeing compared to their younger counterparts [1–5]. Particularly, these studies showed that older participants report less depression and anxiety [1] compared to younger adults, as well as less negative and more positive emotions [2–5].

These studies are in line with the Socioemotional Selectivity Theory (SST) [6] suggesting that older adults tend to be more positive compared to younger counterpart. Indeed, the SST posits that the older population is more motivated to experience positive emotions because they perceive their time ahead as more limited. To maintain this positivity, older people shape their social environments and invest more in closer and satisfying relationships (e.g., with family and friends), since they provide emotionally meaningful interactions [7]. During the COVID-19 pandemic, older adults may have regulated their emotions by focusing on positive emotional states derived from the perception of closeness to family and friends.

On the other hand, the Activity Theory in aging [8] posits that older adults who continue to engage in physical, social, and leisure activities, show enhanced psychological and emotional wellbeing. Recent evidence confirms this theory indicating that, although growing older is associated with a reduction in physical and leisure activities compared to younger adults, older individuals who continue to engage in these activities report higher wellbeing [9]. In addition, the Broaden-and-build theory [10] suggests that leisure engagement can help individuals develop psychological and coping resources to contrast stress, promote positive emotions and reduce the negative ones. Accordingly, the older adults’ greater positivity in stressful conditions may be explained by the fact that they continued or broadened activities during the COVID-19 pandemic, as a kind of coping behavior aimed to protect them from negative life events.

This study examined whether age differences in positive and negative emotional experiences were explained by the engagement in different categories of daily activities and by the perception of more closeness to family and friends during the early lockdown phase of the COVID-19 pandemic in a sample of Italian adults ranging from 18 to 87 years old. Building on previous research conducted during the COVID-19 pandemic (1–5), we expected that increasing age would be associated with more positive and less negative emotional experiences (Hypothesis 1). In addition, in line with SST [6], we expected that age would be positively associated with the perception of more closeness to family and friends (Hypothesis 2). Finally, drawing on SST [6] and Activity Theory in aging [8], we further predicted that the perception of more closeness to family and friends (Hypothesis 3) and engagement in activities (Hypothesis 4) would explain age differences in positive and negative emotional experiences.

## Methods

### Participants

Data were collected between April 9 and May 3, 2020 as a part of a larger study on COVID-19 risk perception [5]. This is a cross-sectional study conducted through a web-based survey on LimeSurvey® in Italian which participants accessed via a link distributed via e-mail and social network messaging during the early stages of COVID-19 outbreak in Italy. Participants were a convenience sample selected based on their accessibility to the online survey.

A total of 1,765 respondents completed the questionnaire under the restrictions that they had to be at least 18 years old and living in Italy during the compilation of the survey. The study was approved by the Ethical Committee of the Department of Brain and Behavioral Sciences of the University of Pavia (no. 46/2020), and informed consent was obtained from each participant.

#### Exclusion criteria

Emotional states and engagement in daily activities during the pandemic were the key variables considered for this study. For this reason, we excluded individuals who could present alterations or limitations in the variables examined, such as healthcare workers (*n* = 108; 6.1%) and participants reporting being diagnosed with COVID-19 (*n* = 8; 0.5%) or experiencing symptoms attributable to it (*n* = 192; 11%). Applying these exclusion criteria, the final sample was composed of 1,457 participants (Table 1).

**Table 1:** Sample characteristics

Characteristics (*n*, %)	Age					
	18–29 (*n* = 243)	30–39 (*n* = 299)	40–49 (*n* = 230)	50–59 (*n* = 255)	60–69 (*n* = 285)	Over 70 (*n* = 145)
Age (*M; *DS)	24.77 (3.11)	34.22 (2.82)	44.44 (2.82)	54.49 (2.83)	64.02 (2.81)	74.41 (3.88)
Gender						
Female	188 (77)	220 (74)	164 (71)	202 (79)	207 (73)	77 (53)
Male	55 (23)	79 (26)	66 (29)	53 (21)	78 (27)	68 (47)
Education						
Not having university degree	100 (41)	116 (390)	141 (61)	171 (67)	179 (63)	83 (57)
Having university degree	143 (59)	183 (61)	89 (39)	84 (33)	106 (37)	62 (43)
Marital status						
Unmarried	194 (80)	90 (30)	73 (32)	77 (30)	69 (24)	39 (27)
Married	49 (20)	209 (70)	157 (68)	178 (70)	216 (76)	106 (73)
Employment						
Not working	119 (49)	20 (7)	14 (6)	38 (14)	154 (54)	110 (76)
Working	124 (51)	279 (93)	216 (94)	218 (86)	131 (46)	35 (24)

Age cohorts were computed for presentation purposes. In the analyses, age was treated as a continuous variable. For the variable Age, the table reports means and (deviation standard). For all other characteristics, the table reports the frequency and (percentage)

### Measures

#### Socio-demographic characteristics

We assessed socio-demographic variables by asking participants’ age, gender (male = 0; female = 1), education (not having university degree = 0; having university degree = 1), marital status (not married = 0; married = 1), employment (not working = 0; working = 1), living alone (0 = no; 1 = yes).

#### Positive and negative emotional experiences

We assessed participants’ positive and negative emotional experiences using the 37-item shortened version of the Profile of Mood States (POMS) [11]. Participants rated, on a 5-point Likert scale (0 = *not at all*; 4 = *extremely*), the extent to which they experienced each emotional state during the last week. POMS yields separate subscales for depression-dejection (e.g., unhappy, sad), tension-anxiety (e.g., anxious, nervous), anger-hostility (e.g., angry, annoyed), fatigue-inertia (e.g., exhausted, weary), confusion-bewilderment (e.g., confused, bewildered), and vigor-activity (e.g., energetic, active). The items of the vigor-activity subscale were summed to create a score of positive emotional experience^1^ (Cronbach’s alpha = 0.87). The scores of the depression-dejection, tension-anxiety, anger-hostility, fatigue-inertia, and confusion-bewilderment were summed to create a negative emotional experience score (Cronbach’s alpha = 0.96). Higher scores indicate high levels of either positive or negative emotional experience.

#### Everyday activities during pandemic

Information on everyday activities that participants engaged during the COVID-19 lockdown was obtained using the open question: “What did you start or re-start doing during COVID-19 lockdown?” Participants’ responses were coded offline to identify the activities started or re-started during the lockdown. Then, activities were grouped into 8 categories following the classification adopted in previous studies [12, 13]: physical activity (e.g., sport, gym, yoga, pilates), cognitive activity (e.g., reading, writing, playing musical instruments, studying something new, playing games, working crossword puzzles), productive activity (e.g., sewing, painting, drawing, model-making, photography), recreational activity (e.g., watching television, listening to the radio, browsing social networks), domestic activity (e.g., clean house, gardening), social activity (e.g., spending time with family and friends, participating in video or phone calls), self-care activity (e.g., relaxing, resting, sleeping, time for oneself), and religious/spiritual activity (e.g., praying, meditating). The dependent variable for each category was coded as 1 when participants engaged in at least one activity in the category or 0 otherwise.

#### Perception of more closeness to relatives and friends during the pandemic

We assessed participants’ perception of more closeness to family (i.e., wife/husband, son, parents) and friends during the pandemic asking participants “Compared to usual, in this period of emergency, I feel closer to family/friends”. For each question (family and friends, respectively), responses were provided on a 5-point Likert scale (0 = not at all; 4 = extremely)*.*

### Statistical analysis

Skewness of frequency distribution was used to judge the normality of data. Data are normally distributed when skewness is equal to zero [14], with values between − 2 and + 2 considered acceptable cut-off [15]. Absolute values of skewness for all our continuous variables fall below 1, indicating that adoption of parametric tests was appropriate. Therefore, we summarized continuous variables through means and standard deviations, and categorical variables through frequencies.

First, correlation analyses were conducted to examine the relationships between age, positive/negative emotional experiences and the other key variables included in the study. We computed Pearson correlations between age, positive/negative emotional experiences, and closeness to family and friends. The correlations of age, positive/negative emotional experiences and other variables are point-biserial correlations reflecting relationships between dichotomous variables and continuous variables. Subsequently, we ran hierarchical regression analyses to test whether age differences in positive and negative emotional experiences were explained by the engagement in daily activities and/or by the perception of more closeness to family and friends. In these regressions, we entered age in the first step, followed by socio-demographic variables in the second step, activities in the third step, and perception of more closeness to family and friends in the final step. Only variables that were significantly associated with age were entered. The assumption of normal distribution of the residuals was checked by inspecting skewness values with acceptable limits of ± 2 [15]. We checked absence of multicollinearity among predictors using *tolerance* statistic greater than 0.2 [14]. The assumption of independent errors was checked using the Durbin–Watson statistic, with values close to 2 meaning that the residuals are uncorrelated [14]. All analyses were conducted using SPSS [16].

## Results

### Sample characteristics

The characteristics of the study sample are summarized in Table 1. The sample included 1,457 participants aged 18–87 years (*M*_age_ = 47.63; SD = 16.34; 73% females). Most participants declared that they were married (63%), were working (69%), and did not have a university degree (54%).

### Correlation analyses

**Fig. 1:**
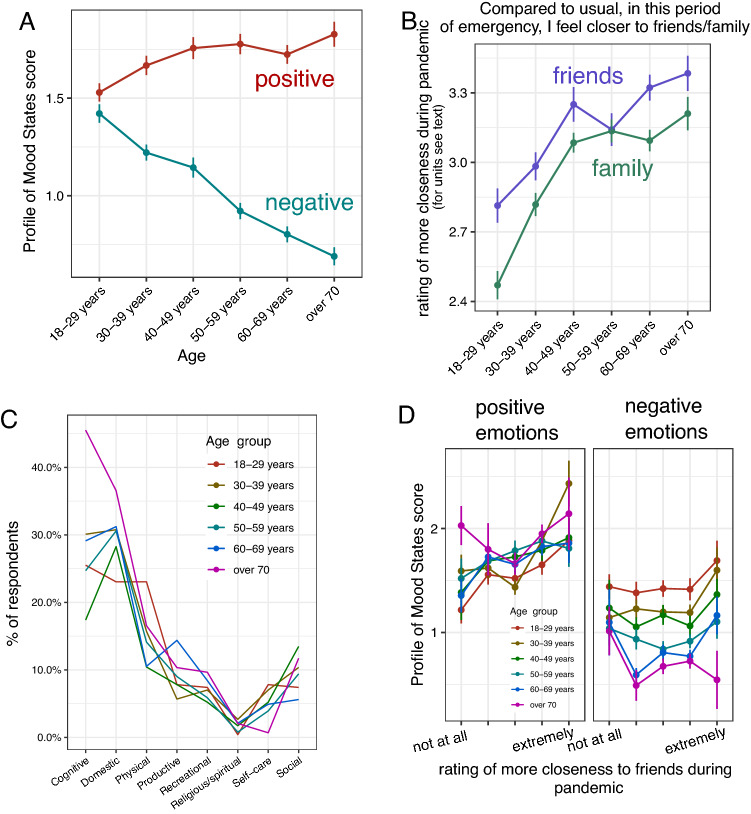
Age differences in emotional experience (**A**), the perception of more closeness to friends and family (**B**), the engagement in daily activities (**C**) during the pandemic, and relationship between perception of more closeness and emotional experience (**D**)*.* Age cohorts were computed for presentation purposes. In the analyses, age was treated as a continuous variable. The figure shows that increasing age was associated with greater positive and less negative emotional experiences (**A**) and with greater perception of closeness to friends and family (**B**). Across ages, participants reported starting or re-starting various categories activities during the pandemic (**C**). Perception of more closeness to friends was associated with greater positive emotional experience across the various age groups (**D**)

The results of the correlation analyses are presented in Table 2. We found that age was positively correlated with positive emotional experience, and negatively with negative emotional experience (see Fig. 1A). Age was negatively associated with gender, education, and employment status, and positively associated with marital status and living alone. Moreover, age was negatively correlated with physical and self-care activities, while it was positively correlated with cognitive, productive, domestic activities (see Fig. 1C), and the perception of more closeness to family and friends (see Fig. 1B).

**Table 2:** Bivariate correlations between age and dependent variables with socio-demographic variables, closeness and activities

	Age	Positive emotional experience	Negative emotional experience
Age	–	0.09***	− 0.32***
Positive emotional experience	0.09***	–	− 0.46***
Negative emotional experience	− 0.32***	− 0.46 ***	–
Gender (female)	− 0.10***	− 0.07**	0.13***
Education (university degree)	− 0.17***	0.04	0.04
Marital status (married)	0.29***	0.11***	− 0.17***
Employment status (working)	− 0.23***	0.07*	0.04
Living alone (yes)	0.14***	0.01	− 0.01
Physical activity (yes)	− 0.08**	0.10***	0.01
Cognitive activity (yes)	0.09***	0.05*	− 0.06*
Productive activity (yes)	0.08**	− 0.01	0.01
Recreational activity (yes)	0.03	− 0.01	0.01
Domestic activity (yes)	0.09***	0.10***	0.04
Social activity (yes)	− 0.01	0.08**	− 0.02
Self-care activity (yes)	− 0.08**	0.05	− 0.01
Religious/Spiritual activity (yes)	0.02	0.06*	0.01
Closeness to family	0.26***	0.09***	− 0.08**
Closeness to friends	0.18***	0.16***	− 0.01

Terms in parentheses denote dichotomous variables coded as 1

*p < 0.05

**p < 0.01

***p < 0.001

Positive emotional experience positively correlated with marital status (higher positive emotional experience was associated with being married), employment status (higher positive emotional experience was associated with working), physical, cognitive, domestic, social, and religious/spiritual activities, and the perception of more closeness to relatives and friends. Positive emotional experience negatively correlated with gender (lower positive emotional experience was associated with female). Negative emotional experience was positively correlated with gender (higher negative emotional experience was associated with female), while negatively correlated with marital status (lower negative emotional experience was associated with being married), cognitive activities and the perception of more closeness to family.

### Regression analyses

Residuals were normally distributed (absolute skewness ≤ 0.95). Tolerance statistic ≥ 0.6 indicated no multicollinearity among predictors. Inspection of scatterplots of the standardized residuals plotted against the standardized predicted values showed no deviations from linearity and no homoscedasticity. Finally, Durbin–Watson statistic reported values 1.9 and 2 for positive and negative emotion, respectively, showing that also the assumption of independent errors was met.

The results of the hierarchical regression analyses are presented in Tables 3 and 4. When positive emotional experience was dependent variable (Table 3), in the first step, age was a significant predictor. When the socio-demographic variables significantly associated with age were added, age remained a significant predictor of positive emotional experience. Next, when daily activities significantly associated with age were added to the model, age remained a significant predictor of positive emotional experience. Finally, when the perception of more closeness to family and friend was added into the model, the association of age and positive emotional experience was no longer significant because of the significant contribution of the variable closeness to friends (Fig. 1D). The variables with a significant contribution to explaining positive emotional experience in the final model were: gender, marital status, employment status, physical, domestic, and self-care activities, and perception of more closeness to friends.

**Table 3:** Hierarchical Regression Analysis on Positive Emotional Experience

Variables	Step 1			Step 2			Step 3			Step 4		
	*B*	*SE*	*β*	*B*	*SE*	*Β*	*B*	*SE*	*β*	*B*	*SE*	*β*
Age	0.00	0.00	0.10***	0.00	0.00	0.08*	0.00	0.00	0.08**	0.00	0.00	0.05
Gender (female)				− 0.13	0.05	− 0.07**	− 0.17	0.05	− 0.09***	− 0.20	0.05	− 0.11***
Marital status (married)				0.16	0.06	0.10**	0.16	0.06	0.10**	0.16	0.06	0.10**
Education (university degree)				0.07	0.04	0.04	0.05	0.04	0.03	0.05	0.04	0.03
Employment status (working)				0.12	0.05	0.07*	0.13	0.05	0.07*	0.11	0.05	0.06*
Living alone (yes)				0.11	0.08	0.04	0.10	0.08	0.04	0.09	0.08	0.03
Physical activity (yes)							0.26	0.06	0.12***	0.25	0.06	0.11***
Cognitive activity (yes)							0.00	0.05	0.00	− 0.02	0.04	− 0.35
Productive activity (yes)							0.01	0.08	0.00	0.01	0.07	0.00
Domestic activity (yes)							0.13	0.05	0.07**	0.12	0.05	0.07**
Self− care activity (yes)							0.24	0.09	0.07*	0.21	0.09	0.06*
Closeness to family										0.02	0.03	0.03
Closeness to friends										0.10	0.02	0.14***
Change in *R*^2^	0.01***			0.02***			0.02***			0.02***		

Terms in parentheses denote dichotomous variables coded as 1

*p < 0.05

**p < 0.01

***p < 0.001

**Table 4:** Hierarchical regression analysis on negative emotional experience

Variables	Step 1			Step 2			Step 3			Step 4		
	*B*	*SE*	*β*	*B*	*SE*	*Β*	*B*	*SE*	*β*	*B*	*SE*	*β*
Age	− 0.01	0.00	− 0.33***	− 0.01	0.00	− 0.31***	− 0.01	0.00	− 0.31***	− 0.01	0.00	− 0.32***
Gender (female)				0.15	0.04	0.09***	0.16	0.04	0.10***	0.15	0.04	0.09***
Marital status (married)				− 0.13	0.05	− 0.08**	− 0.13	0.05	− 0.08**	− 0.13	0.05	− 0.08**
Education (university degree)				− 0.06	0.04	− 0.04	− 0.06	0.04	− 0.04	− 0.06	0.04	− 0.04
Employment status (working)				− 0.05	0.04	− 0.03	− 0.05	0.04	− 0.03	− 0.05	0.04	− 0.03
Living alone (yes)				0.06	0.07	0.02	0.06	0.07	0.03	0.06	0.07	0.02
Physical activity (yes)							− 0.08	0.05	− 0.04	− 0.08	0.05	− 0.04
Cognitive activity (yes)							0.00	0.04	0.00	− 0.00	0.04	− 0.00
Productive activity (yes)							0.01	0.07	0.00	0.01	0.07	0.00
Domestic activity (yes)							− 0.11	0.04	− 0.01	− 0.01	0.04	− 0.01
Self-care activity (yes)							− 0.17	0.08	− 0.05*	− 0.18	0.08	− 0.05*
Closeness to family										0.01	0.02	0.01
Closeness to friends										0.03	0.02	0.04
Change in *R*^2^	0.11***			0.02***			0.00			0.00		

Terms in parentheses denote dichotomous variables coded as 1

^*^p < 0.05

^**^p < 0.01

^***^p < 0.001

With negative emotional experience as dependent variable (Table 4), in the first step, age was a significant predictor. The association between age and negative emotional experience remained significant even when we added in the second step the socio-demographic variables that were significantly associated with age. However, when the other variables significantly associated with age were added in consecutive steps to the model, they did not lead to a significant increase in variance explained.

## Discussion

The present study investigated positive and negative emotional experiences across the lifespan in adulthood during the COVID-19 lockdown in Italy and whether the engagement in daily activities and the perception of more closeness to family and friends during the pandemic explained age-related differences [6, 8].

Consistent with recent empirical research during COVID-19 pandemic [1–5], we found that higher age was significantly associated with more positive and less negative emotional experiences (Hypothesis 1 supported). This result could be because older adults are able to manage and regulate their emotions better than younger adults [17], even in a situation in which they are the most at risk for mortality if they would contract COVID-19 [18].

Looking at the association between positive emotional experience and the perception of more closeness to family and friends during the pandemic, older adults reported feeling more closeness to friends and family compared to what younger individuals have felt (Hypothesis 2 supported). However, age differences in positive emotional experience were explained by the perceived of more closeness to friends (Hypothesis 3 supported). This pattern of results is consistent with the SST [6]: the more positive emotion experienced by relatively older adults was due to the feeling of more closeness relationships with friends of their social network. Our results support theory and empirical evidence showing that, even in a stressful situation, older people feel closer to family and friends compared to younger adults, and that the positive emotional experience derived from the closeness to friends promotes their wellbeing [6, 7]. The benefit in older ages of closeness to friends compared to those provided by closeness to family is not unexpected. Indeed, previous studies reported that, in older adults, carrying out activities with friends increased more positive effect and psychological wellbeing compared to performing activity with family [19, 20]. Since friends who remain in the older adults’ social network are selected in accordance with older adults’ emotional needs [6], and they may be able to provide more emotional support than family [21], it may be that older adults were more likely to share concerns regarding the pandemic with friends than with family members. This may have contributed to increasing their positive emotional experience during the pandemic lockdown.

Regarding the association between positive emotional experience and the engagement in daily activities during the pandemic, even though older adults engaged more in cognitive, productive, and domestic activities compared to younger counterparts, our results showed that age differences in positive emotional experience were not accounted by the level of engagement in these activities (Hypothesis 4 not supported). This result is in contrast with our expectations and suggests that older adults’ positive emotional experience was not derived from the engagement in daily activities, as would have been suggested by the Activity Theory of aging [8]. We may speculate that in older adults, the emotions coming from the experience of closeness to friends were more important than the benefits derived from the engagement in activities. Alternatively, we may speculate that, due to the restrictions imposed by the lockdown, participants carried out activities less frequently than usual and this may explain why age differences in positive emotional experience were not accounted by the level of engagement in daily activities.

Regarding the negative emotional experience, age differences remained after accounting for the perception of closeness to family and friends and daily activities. This result seems to suggest that in the dimension of negative feelings, older adults regulate their emotion minimizing the potential for negative affect [6] independently from the perception of more closeness to family and friends or the benefits derived from the engagement in daily activities. It is worth noting that there were still age differences in both positive and negative emotional experience even after accounting for socio-demographic variables. These findings suggest that age-related differences in demographic variables do not explain more positive and less negative emotional experiences in aging, as was also reported in previous research on COVID-19 [1, 2].

The strength of the present study is to have evaluated the role of the perception of closeness to family and friends, as a relevant factor to promote emotional wellbeing according to the SST [6]. This factor was found to be crucial for older adults’ more positive emotional experience, but not for negative emotional experience. It may be that in the face of the pandemic stressful situation, older people’s fewer negative reactions, compared to younger adults, are due to their more effective emotional regulation, which was previously shown to be an intrinsic characteristic of growing old [6, 17].

Our investigation had limitations. First, our data were cross-sectional in nature. Hence, our study does not allow the observation of changes in positive and negative emotional experiences over time and age differences could therefore be explained by a range of non-age variables such as the historical period during which participants grew up. Second, we recruited a convenience sample which is not necessarily representative of the entire population. As well as the study did not consider the effect of pandemic and lockdown on emotional experience in specific populations of older adults, such as those with low social economic status, with neurodegenerative diseases, or living in nursing home, who could experience more distress due to the actual situation. Moreover, given the correlational nature of the present study, it remains unclear whether closeness to friends caused positive emotional state or the other way around. However, it could also be imagined that those who already have a more positive emotional state would find it easier to connect with friends from that state. Future studies could use longitudinal designs to decide between these two possibilities. Finally, the study has been conducted during the early stage of the pandemic outbreak; hence, it could be interesting to see whether older adults’ emotional state remained stable during the progression of pandemic.

Since our findings support the importance of closeness, to cope with stressful situations, such as that of pandemic, it is important to encourage older adults to increase the closeness to their social network.

## Data Availability

The dataset analyzed in the current study is freely available on the OSF repository, https://osf.io/ywcmg/?view_only=6b1984e47bdd40838b073512e5d5f4cc.

## Footnotes

1 We exclude the item “in a good mood” because it was the only item of the vigor-activity subscale negatively correlated with age (*r* = − 0.11, *p* =  < 0.001).
